# ﻿*Amolops cuongi* (Amphibia, Anura, Ranidae), a new species from the Hoang Lien Range, Vietnam

**DOI:** 10.3897/zookeys.1256.158846

**Published:** 2025-10-22

**Authors:** Anh Van Pham, Chung Van Hoang, Benjamin Tapley, Luan Thanh Nguyen, Hanh Huu Nguyen, Toi Van La, Thomas Ziegler, Jodi J. L. Rowley, Truong Quang Nguyen, Minh Duc Le

**Affiliations:** 1 Faculty of Environmental Sciences, University of Science, Vietnam National University, Hanoi, 334 Nguyen Trai Road, Hanoi, Vietnam; 2 Institute of Biology, Vietnam Academy of Science and Technology, 18 Hoang Quoc Viet Road, Hanoi 10072, Vietnam; 3 Green Environment Centre, 119, Lane 3, Tho Lao Street, Hanoi, Vietnam; 4 Zoological Society of London, Regent’s Park, London, NW1 4RY, UK; 5 Asian Turtle Program of Indo-Myanmar Conservation, R.1301, CT1 Bac Ha C14 Building, To Huu Str., Hanoi, Vietnam; 6 Hoang Lien National Park, 89 Nguyen Chi Thanh Street, Sa Pa, Lao Cai Province, Vietnam; 7 AG Zoologischer Garten Köln, Riehler Strasse 173, D–50735 Cologne, Germany; 8 Institute of Zoology, University of Cologne, Zülpicher Strasse 47b, D–50674 Cologne, Germany; 9 Australian Museum Research Institute, Australian Museum, 1 William St, Sydney, NSW, 2010, Australia; 10 Centre for Ecosystem Science, School of Biological, Earth and Environmental Sciences, University of New South Wales, Sydney NSW 2052, Australia; 11 Graduate University of Science and Technology, Vietnam Academy of Science and Technology, 18 Hoang Quoc Viet Road, Hanoi 10072, Vietnam; 12 Central Institute for Natural Resources and Environmental Studies, Vietnam National University, Hanoi, 19 Le Thanh Tong, Hanoi, Vietnam; 13 Department of Herpetology, American Museum of Natural History, Central Park West at 79; 14 th; 15 Street, New York, New York 10024, USA

**Keywords:** *Amolops mantzorum* group, genetically distinct, molecular phylogenetics, morphology, taxonomy

## Abstract

A new species of the genus *Amolops* is described from the Hoang Lien Range, northwestern Vietnam as *Amolops cuongi***sp. nov.** While morphological and molecular data assign these individuals to the *Amolops mantzorum* group, the new species is distinguishable from its congeners on the basis of a combination of the following diagnostic characters: size small (SVL 33.9–36.9 mm in males; 37.9–44.4 mm in females); head longer than wide; vomerine teeth absent or weakly developed; snout short (SE/SVL 0.15–0.17 in males; 0.14–0.16 in females); tympanum small (TD/ED 0.26–0.37 in males; 0.25–0.35 in females); the absence of circummarginal groove on the first finger; disc of finger III larger than tympanum; supratympanic fold present; dorsolateral fold absent; webbing formula I0–1II0–1III0–1IV1–0V; the presence of a band of small spinules and/or tubercles running from below nares, along upper lip, around lower half of eye, between tympanum and eye and rear axis of mandibles; granular skin on flanks and ventral surfaces of body; in life, dorsal body colouration of dark brown with diffuse-edged blotches of bluish grey, copper and yellowish green or pale green and copper; ventral surface of throat, chest and belly pale cream with white dots; males without vocal sacs; and nuptial pad velvety without spines. In the phylogenetic analysis using a combination of mitochondrial 16S ribosomal RNA, ND2, and cytochrome *b* (cyt *b*) genes, the new species is strongly supported as the most genetically distinct member of the *Amolops mantzorum* group with genetic distance ≥ 1.53% in the 16S rRNA, ≥ 8.70% in ND2, and ≥ 8.56% in cyt *b* compared to other members within the genus *Amolops*.

## ﻿Introduction

The genus *Amolops* Cope, 1865 currently contains 87 recognized species distributed in Asia from Nepal and northern India eastwards to China and southwards to Malaysia (Frost 2025). Recent studies assigned the members of this genus into ten species groups, namely the *Amolops chayuensis* group, *A. daiyunensis* group, *A. hainanensis* group, *A. laurentis* group, *A. mantzorum* group, *A. marmoratus* group, *A. monticola* group, *A. ricketti* group, *A. spinapectoralis* group, and *A. viridimaculatus* group (Jiang et al. 2021; Liu et al. 2024).

Morphologically, the members of the *Amolops mantzorum* group are characterised by the absence of dorsolateral folds (glandular dorsolateral folds may be present), circummarginal groove on the disc of the first finger, and tarsal fold and tarsal glands (Fei et al. 2005, 2009; Tang et al. 2023; Qian et al. 2023). Fei et al. (2009) listed five species in the *Amolops mantzorum* group, namely *A. granulosus* (Liu & Hu), *A. lifanensis* (Liu), *A. loloensis* (Liu), *A. mantzorum* (David), and *A. viridimaculatus* (Jiang). Lu et al. (2014) suggested that the *Amolops mantzorum* species group consists of four well-recognised species, i.e., *A. granulosus*, *A. lifanensis*, *A. loloensis*, and *A. viridimaculatus*, along with five putative species, comprising three disputable species, *Amolops kangtingensis* Inger, *A. jinjiangensis* (Su, Yang & Li) and *A. tuberodepressus* Liu & Yang, and the nominal species *A. mantzorum*, which may in fact consist of two cryptic species (Lu et al. 2014). Fei et al. (2017) described a new species of this group, *A. xinduqiao* Fei, Ye, Wang & Jiang, and considered *A. kangtingensis* a synonym of *A. mantzorum*. However, Dufresnes and Litvinchuk (2022) subsequently regarded *A. xinduqiao* as a subspecies of *A. mantzorum*. In addition, Zeng et al. (2020) and Wu et al. (2020) removed *Amolops viridimaculatus* from the *A. mantzorum* group and placed it in a separate group. Recently, Liu et al. (2024) synonymised *A. ottorum* with *A. minutus* based on morphological and molecular evidence. Currently, the *A. mantzorum* group contains eleven species, comprising *A. ailao* Tang, Sun, Liu, Luo, Yu & Du; *Amolops dafangensis* Li, Liu, Ke, Cheng & Wang; *A. granulosus* (Liu & Hu); *A. jinjiangensis* Su, Yang & Li; *A. lifanensis* (Liu); *A. loloensis* (Liu); *A. mantzorum* (David); *A. minutus* Orlov & Ho; *A. sangzhiensis* Qian, Xiang, Jiang, Yang & Gui; *A. shuichengicus* Lyu & Wang; and *A. tuberodepressus* Liu & Yang (Zeng et al. 2020; Dufresnes and Litvinchuk 2022; Tang et al. 2023; Liu et al. 2024).

During our recent field work in northern Vietnam, specimens of *Amolops* were collected from the Hoang Lien Range in Lai Chau and Lao Cai provinces. These specimens were identified as an unnamed taxon of the *A. mantzorum* species group based on molecular and morphological data. Therefore, we herein describe it as a new species to science, *Amolops cuongi* sp. nov.

## ﻿Materials and methods

### ﻿Sampling

**Figure 1.**
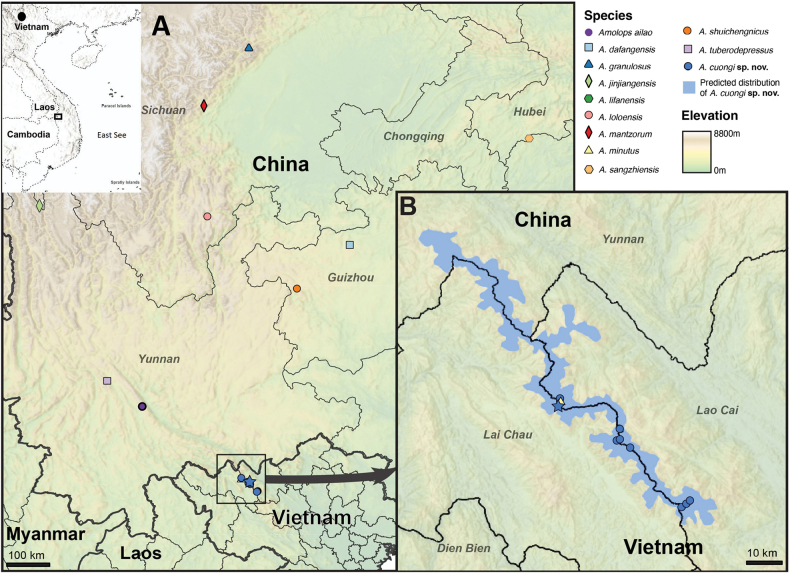
A. Map of the type localities of species in the *Amolops mantzorum* group; B. Zoomed in area showing the known localities and predicted distribution of *Amolops cuongi* sp. nov. Star indicates type locality of *Amolops cuongi* sp. nov. The precise type locality of *A. lifanensis* is not known (Lishan and Maoxian counties in central Sichuan Province).

Specimens were collected at night in forest habitats in the Hoang Lien Range, northern Vietnam between September 2017 and September 2020 (Fig. 1). The geographical coordinates were recorded using GPS Garmin 62s and Garmin GPSMAP 64CSx GPS receiver. Coordinates were recorded as latitude and longitude in decimal degrees and referenced to the World Geodetic System of 1984 (WGS84). Specimens were collected by hand from 19:00 to 22:00 and from 04:30 to 05:30. Specimens were photographed in life before being euthanised using a 20% solution of benzocaine applied to the ventral surface of the frog. Tissue samples (liver) for molecular analyses were extracted from freshly euthanised specimens and stored in absolute ethanol prior to the fixation of specimens with 10% formalin or fixed in 85% ethanol and subsequent storage in 70% ethanol.

Voucher specimens were subsequently deposited in the collection of the Institute of Biology (**IB**, formerly known as Institute of Ecology and Biological Resources – IEBR), Hanoi, Vietnam, the Institute of Institute of Life Science– Herpetology (**ILS H**), Ho Chi Minh City, Vietnam. A referred specimen was deposited at the Hoang Lien National Park headquarters (**HLNP**) as a reference for national park scientists.

### ﻿Molecular analysis

Tissue samples were extracted using PureLink RNA Micro Scale Kit (Thermo Fisher Scientific company), following the manufacturer’s instructions. Genomic DNA was amplified using an Applied Biosystems PCR machine. The PCR total volume was 25 μl, consisting of 12 μl of mastermix, 6 μl of water, 1 μl of each primer at a concentration of 10 pmol/μl, and 5 μl of DNA. Primers used in the PCR and sequencing were as follows: the primer pair, LR N 13398 (5’-CGCCTGTTTACCAAAAACAT- 3’; forward), LR J 12887 (5’-CCGGTCTGAACTCAGATCACGT-3’; reverse) (Simon et al. 1994) was used to amplify a fragment of the mitochondrial 16S rRNA gene; the primer pair Met-LND2 (5’-CAATGTTGGTTAAAATCCTTCC-3’), and Trpe-HND2 (5’-AGGCTTTGAAGGCCTTTGGTC-3’) (Stuart et al. 2006), was used to amplify a fragment of the NADH dehydrogenase subunit 2 (ND2) gene; and the primer pair AmF1 (5’-TCTCATCCTGATGAAACTTTGGCTC-3’) and AmR3 (5’-CTACTGGTTGTCCTCCGATTCATGT-3’) (Lu et al. 2014) was used to amplify a fragment of the cytochrome *b* (cyt*b*) gene. PCR products were sent to Apical Scientific (Malaysia) (https://apicalscientific.com) for sequencing. The obtained sequences were deposited in GenBank (Suppl. material 1: table S1).

In addition to sequences generated for six samples of the new population from Lai Chau and Lao Cai provinces, we used 16S rRNA, ND2, and cyt*b* data of 35 samples belonging to species within the *Amolops mantzorum* group available from GenBank for phylogenetic analyses. Sequences of *Amolops* species outside the *A. mantzorum* group and *Odorrana jingdongensis* Fei, Ye & Li were included in the analysis as the outgroups (Wu et al. 2020). Localities and accession numbers of all sequences used in the study can be found in Suppl. material 1: table S1.

Chromas Pro software (Technelysium Pty Ltd., Tewantin, Australia) was used to edit the sequences, which were then aligned using ClustalX (Thompson et al. 1997) as embedded in MEGA11 (Tamura et al. 2021) with default parameters and subsequently optimised manually in BioEdit 7.0.5.2 (Hall 1999). Pairwise comparisons of uncorrected sequence divergence (p distance) were calculated using MEGA11 (Tamura et al. 2021). Variance was estimated using bootstrap method with 1000 replicates using nucleotide substitution while gap/missing data were treated via pairwise deletion.

Prior to Bayesian analyses, the optimum nucleotide substitution models for 16S rRNA, ND2 and cyt*b* partitions were selected using Kakusan 4 (Tanabe 2011), based on the Akaike information criterion (AIC). Bayesian inference (BI) was estimated using MrBayes v. 3.2 (Ronquist et al. 2012) and GTR + G model. Two independent runs of four Markov Chains, three heated and one cold, were performed for 10,000,000 generations. Tree was sampled every 100 generations, and a consensus topology was calculated using 70,000 trees after discarding the first 30000 trees (burnin = 3,000,000). We checked parameter estimates and convergence using Tracer v. 1.7.1 (Rambaut et al. 2018). For maximum likelihood (ML) analysis, IQ-TREE v. 1.6.12 (Nguyen et al. 2015) along with GTR+F+I+G4 model was used with 10,000 ultrafast bootstrap replications (UFB) (Hoang et al. 2018). We considered Bayesian posterior probability (BPP) and ultrafast bootstrap (UFB) support values of greater than or equal to 0.95 for BPP and 95% for UFB as strong support for a clade (Ronquist et al. 2012; Hoang et al. 2018).

### ﻿Morphological analysis

Measurements were taken from 19 preserved specimens using a digital caliper to the nearest 0.1 mm; morphometrics followed Orlov and Ho (2007), Stuart et al. (2010), and Pham et al. (2019):

**SVL** snout-vent length (from tip of snout to cloaca);

**HL** head length (from the back of mandible to tip of snout);

**HW** maximum head width (across angles of jaws);

**SE** distance from tip of snout to anterior corner of eye;

**SND** distance from nostril to the tip of snout;

**END** eye to nostril distance (from anterior corner of eye to the nostril);

**IND** internarial distance (distance between nostrils);

**IOD** interorbital distance (minimum distance between upper eyelids);

**ED** horizontal eye diameter (from the anterior corner to the posterior corner of the eye);

**TD** maximum tympanum diameter;

**TED** tympanum-eye distance (from anterior margin of tympanum to posterior corner of the eye);

**HND** Hand length (from base of palm to tip of third finger);

**FTD** maximum width of disc of finger III;

**FL** femur length (from vent to knee);

**TL** tibia length (from knee to tarsus);

**FOT** foot length (from proximal edge of inner metatarsal tubercle to tip of fourth toe); and

**HTD** maximum width of disc of fourth toe.

All measurements were taken from the right side of the specimen and by the first, second, and fourth author for consistency. Interdigital toe webbing formula follows Savage and Heyer (1997). Sex and maturity were determined by gonadal inspection and the presence of nuptial pads.

### ﻿Species distribution mapping

An estimated species distribution map (Fig. 1) was created in ArcGIS Pro3.5 (Esri, California, USA). The species’ distribution was generated using the International Union for Conservation of Nature (IUCN) elevation raster (IUCN SSC 2017). The range for the newly described species was estimated by clipping the elevation to above 1900 m a.s.l. Areas of habitat were deemed suitable and included in maps if they are within species’ estimated elevation range, and are not separated from known localities by any continuous stretch of unsuitable habitat with a distance equal to or greater than 1 km. Extent of occurrence (EOO), defined as the area of a minimum convex polygon that passes all known and inferred sites occupied by the species, was measured using the EOO Calculator for ArcGIS Pro (Toolbox v. 2.0).

## ﻿Results

### ﻿Phylogenetic analyses

The combined matrix of 16S rRNA, ND2, and cyt*b* contained 2296 aligned characters. In terms of pairwise genetic distance based on 16S rRNA data, interspecific uncorrected *p*-distance of the *Amolops mantzorum* group ranged from 0.19% (between *A. jinjiangensis* and *A. sangzhiensis*) to 6.75% (between *A. lifanensis* and *A. ailao*) (Suppl. material 1: tables S2–S4). The genetic divergence between the new form from Vietnam and its congeners ranged from 1.35% (*A. shuichengicus*) to 5.14% (*A. lifanensis*) (Suppl. material 1: table S2). In the ND2 gene, interspecific uncorrected *p*-distance of the *Amolops mantzorum* group ranged from 3.99% (between *A. loloensis* and *A. jinjiangensis*) to 19.36% (between *A. loloensis* and *A. lifanensis*). The genetic divergence of the new form from Vietnam and its congeners varied from 8.70% (*A. minutus*) to 19.14% (*A. lifanensis*) (Suppl. material 1: table S3). In the cyt*b* gene, interspecific uncorrected *p*-distance of the *Amolops mantzorum* group ranged from 5.04% (between *A. loloensis* and *A. jinjiangensis*) to 11.53% (between *A. mantzorum* ssp. and the new form). The genetic divergence of the new form from Vietnam and its congeners ranged from 7.80% (*A. loloensis*) to 11.53% (*A. mantzorum* sp.) (Suppl. material 1: table S4).

**Figure 2.**
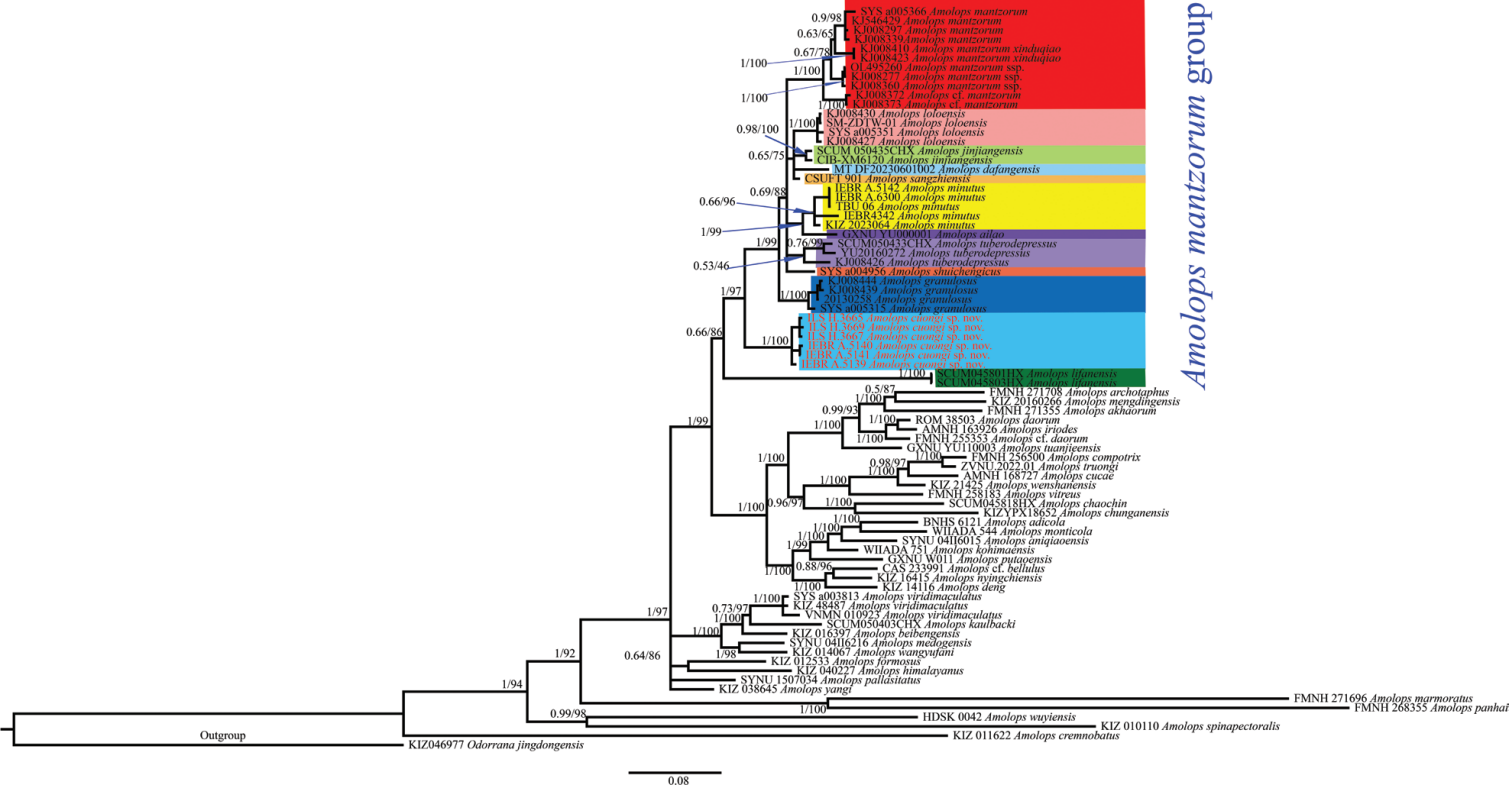
Bayesian phylogeny based on 16S, ND2, and cyt*b* genes. Numbers on branches are BPP and UFB, respectively.

Phylogenetic analyses employing ML and BI methods were nearly identical, with most well-supported nodes on the ML tree also well-supported on the BI tree, and only the BI tree is presented in Fig. 2. The new form is strongly supported as a member of the *A. mantzorum* group and as a sister clade to all remaining species of the *A. mantzorum* group (BPP = 1, UFB = 97), except for *A. lifanensis*, while the latter was weakly recovered as a taxon within the species group (BPP = 0.66, UFB = 86) (Fig. 2).

In the following, based on distinct genetic divergence in concert with diagnostic morphological differences compared to their congeners, we describe the newly discovered population of *Amolops* from Lai Chau and Lao Cai province as a species new to science.

### ﻿Species description

#### ﻿ Amolops cuongi sp. nov.

Taxon classificationAnimaliaAnuraRanidae

22ADAF93-5CB2-508D-BC89-0A645C4F5A6C

https://zoobank.org/E3F15F15-12DE-44F1-BC6A-37579E18E48A

Figs 3, 4, 5, 6

##### Type material.

***Holotype*.**IEBR A.5139 (Field No. LC2020.82), • adult male, collected by C. T. Pham, C. V. Hoang, T. V. Phan, N. B. Sung, and A. V. Pham on 16 May 2020, found in evergreen forest near Ho Thau Village, Ho Thau Commune, Lai Chau Province, Vietnam (22.408313°N, 103.608094°E, at an elevation of 2442 m). ***Paratypes*.** In **Lai Chau Province**, Vietnam: • two adult females, IEBR A.5140 (Field No. LC2020.179), IEBR A.5141 (Field No. LC2020.181), the same data as the holotype. • one adult male ILS H.3665 (Figs 5, 6A) and one adult female ILS H.3666^nd^, collected in disturbed evergreen forest of Hoang Lien Range in (22.3473°N, 103.77226°E; 1928 m a.s.l.), on 10 September 2018 by L. T. Nguyen, C. T. Nguyen, and H. V. Luong. In **Lao Cai Province**, Vietnam: • specimens collected in the forest of Hoang Lien Range, near Sa Pa by L. T. Nguyen, C. T. Nguyen, B. Tapley and L. Harding: • one adult female ILS H.3662 (Fig. 6E) and one subadult ILS H.3663 (Fig. 6G) (22.31517°N, 103.76897°E; 2629 m a.s.l.) on 14 September 2017; • one adult female ILS H.3664 (22.31488°N, 103.76845°E; 2690 m a.s.l.) on 14 September 2017; collected by L. T. Nguyen, C. T. Nguyen and L. V. Hoang on 13 June 2018: • one adult male ILS H.3667 (22.3149°N, 103.7686°E; 2635 m a.s.l.) and one adult female ILS H.3668 (22.3145°N, 103.7675°E; 2685 m a.s.l.); • one adult female ILS H.3671 (22.31394°N, 103.76561°E; 2784 m a.s.l.), collected on 19 June 2019 by L. T. Nguyen and C. T. Nguyen; One adult male ILS H.3674 and one subadult ILS H.3675 (22.31394°N, 103.76561°E; 2784 m a.s.l.), collected on 8 September 2019 by C. T. Nguyen; • one adult female ILS H.3666 (Fig. 6B) (22.3473°N, 103.77226°E; 1928 m a.s.l.) collected on 10 September 2018 by L. T. Nguyen, C. T. Nguyen, and L. V. Hoang; • one subadult ILS H.3669 (22.9474°N, 103.8030°E; 2578 ma.s.l.), collected on 14 September 2018 by L. T. Nguyen, C. T. Nguyen, and H V. Luong; • three adult males ILS H.3683 (Fig. 6C), ILS H.3685 and ILS H.3686 and two adult females ILS H.3673 and ILS H.3684 (Fig. 6D) (22.14565°N, 103.96281°E; 2321 m a.s.l.), one adult female ILS H.3672 (22.14087°N, 103.95631°E; 2311 m a.s.l.), collected in undisturbed evergreen forest on Mount Nam Kang Ho Tao, Sa Pa on 13 September 2020 by C. T. Nguyen, G. T. Hoang and Q. L. Tan; • one adult male ILS H.3670 collected in disturbed evergreen forest on Mount Pu Ta Leng, Bat Xat (22.4263°N, 103.61322°E, 2362 m a.s.l.) on 22 March 2018 by C. T. Nguyen, L. T. Nguyen, B. Tapley and C. Portway.

##### Referred specimen.

One subadult HLNP2017 1409 00030 (Fig. 6F) found in bamboo forest on Mount Fansipan, Sa Pa, Lao Cai Province, Vietnam (N22.31483, E103.76883; 2625 m a.s.l.) on 14 September 2017 by L. T. Nguyen, C. T. Nguyen, B. Tapley, and L. Harding. This specimen is not included in the type series due to it being deposited in a local collection. The taxonomic identity of the specimen is not in question.

##### Diagnosis.

*Amolops cuongi* sp. nov. from the Hoang Lien Range is assigned to the *A. mantzorum* species group based on the absence of a dorsolateral fold and the absence of a circummarginal groove on the first finger (Fei et al. 2009, 2017). The new species is also supported as a member of the *A. mantzorum* group based on the molecular analyses (Fig. 2).

*Amolops cuongi* sp. nov. is distinguishable from its congeners by a combination of the following morphological characteristics: (1) size small (SVL 33.9–36.9 mm in males; 37.9–44.4 mm in females); (2) head longer than wide; (3) vomerine teeth absent or weakly developed; (4) snout short (SE/SVL 0.15–0.17 in males; 0.14–0.16 in females); (5) tympanum small, round (TD/ED 0.24–0.37 in males; 0.26–0.35 in females); (6) the absence of a circummarginal groove on the first finger; (7) width of disc of finger III larger than tympanum; (8) supratympanic fold present; (9) dorsolateral fold absent; (10) webbing formula I0–1II0–1III0–1IV1–0V; (11) the presence of a band of small spinules and / or tubercles running from below nares, along upper lip, around lower half of eye, between tympanum and eye and rear axis of mandibles; (12) granular skin on flanks and ventral surfaces of body; (13) in life, dorsal body colouration of dark brown with diffuse-edged blotches of bluish grey, copper and yellowish green or pale green and copper; (14) ventral surface of throat, chest and belly pale cream with white dots; (15) males without vocal sacs; and (16) nuptial pad velvety without spines.

##### Description of holotype.

Adult male; SVL 36.0 mm; head broad and flat (HD/SVL 0.14), longer than wide (HL 12.2 mm, HW 11.8 mm, HL/SVL 0.34, HW/SVL 0.33); snout round anteriorly in dorsal view (SE/SVL 0.15), projecting beyond lower jaw; snout length greater than eye diameter (SE 5.0 mm, ED 5.3 mm); nostril lateral, closer to eye the than to snout tip (SND 3.0 mm, END 2.3 mm); canthus rostralis distinct; loreal region slightly concave; eyes large (ED/HL 0.43, ED/SE 1.0); pupil horizontally oval; internarial distance as wide as interorbital distance (IND 4.2 mm, IOD 4.2 mm), larger than upper eyelid width (UEW 2.9 mm); tympanum distinct, round, small (TD 1.5 mm, TD/ED 0.28); vomerine teeth absent; tongue cordiform, notched posteriorly; body gracile.

***Fore limbs*** robust; relative finger lengths: I<II<IV<III; fingers without webbing; tips of fingers expanded into discs, II–IV with circummarginal grooves; tip of first finger smaller, without circummarginal groove; width of disc of finger III greater than the diameter of tympanum (TD 1.5 mm, FTD 2.5 mm, TD/FTD 0.60); subarticular tubercles at base of fingers II–IV round, others indistinct; inner metatarsal tubercle oval, elongate, indistinct; outer metatarsal tubercle absent, finger I with a large nuptial pad, elongate, ~2/3 the length of finger.

***Hind limbs*** long; tibia longer than thigh (FL 17.9 mm, TL 20.2 mm); relative toe length I<II<III<V<IV; tips of toes expanded into discs; width of disc of toe IV narrower than that of finger III (FTD 2.5 mm, HTD 1.6 mm); webbing formula I0–1II0–1III0–1IV1–0V; subarticular tubercles oval, formula 1, 1, 2, 3, 2; inner metatarsal tubercle elongate; outer metatarsal tubercle absent; tibiotarsal articulation reaches the nostril.

##### Skin texture in life.

Dorsal surface of head, body, and limbs smooth; posterior surface of thighs and ventral surfaces of shanks smooth; flanks, throat, chest, abdomen and ventral surfaces of forearms and thighs weakly granular with the granules largest on flanks; supratympanic folds present; dorsolateral folds absent; band of small spinules present running from below nares, along upper lip, around lower half of eye, between tympanum and eye and rear axis of mandibles, small spinules are largest between tympanum and eye and at rear axes of mandibles; tympanum smooth; limbs smooth on dorsal and ventral surfaces; humeral glands absent.

##### Colouration in life.

Dorsum green with some dark brown spots and some large pale green markings; dorsal surface of head and body with some irregular small black dots; lips pale green; loreal region with a dark brown longitudinal stripe, tip of snout green with small brown dots; around iris white; tympanum dark brown; dorsal surface of fore and hind limbs pale green with dark crossbars; flanks green; ventral surface of throat, chest and belly pale cream with white dots; ventral surface of fore and hind limbs pale brown with some greenish dots; back of the thigh brown with pale dots; plantar aspect of feet brown.

##### Colouration in preservative.

Dorsum and flanks brown with some large grey spots; lips brown; tympanum black; dorsal surface of fore and hind limbs brown with dark crossbars; throat and chest grey; belly grey-brown with pale dots; ventral surface of fore and hind limbs pale grey with pale dots; back of the thigh brown with grey dots; plantar surface of the foot brown.

##### Sexual dimorphism.

**Figure 3.**
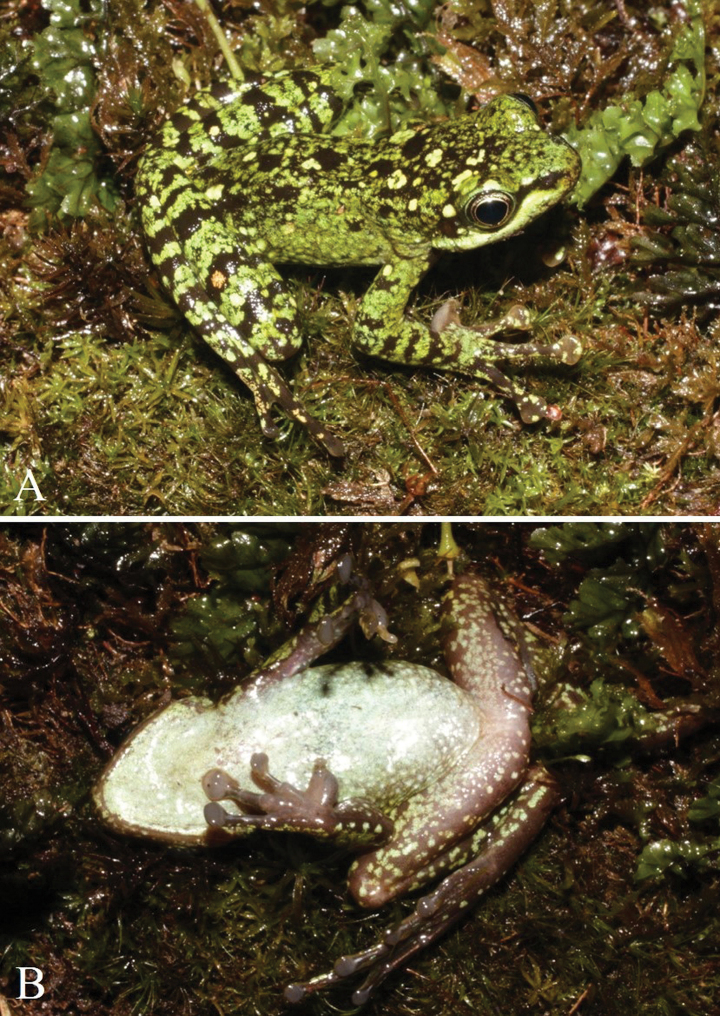
A. Dorsolateral view and; B. Ventral view of the male holotype (IEBR A. 5139) of *Amolops cuongi* sp. nov. in life.

All adult males with a cream-coloured, velvety nuptial pad along base of finger I in preservative (Fig. 5D), greyish brown in life (Fig. 3A, B). Four gravid females (ILS H.3684, ILS H.3662, ILS H.3664, ILS H.3672); Eggs counted and measured in ILS H.3684 only: 44 yellow, round eggs ~3.2–3.4 mm in diameter (*n* = 10 eggs). The presence of white spinules below nares, along upper lip, around lower half of eye, between tympanum and eye and rear axes of mandibles is present in both sexes. Body size of females greater than body size of males (SVL of adult males 33.9–36.9 mm, *n* = 8; adult females 37.9–44.4 mm, *n* = 7).

**Figure 4.**
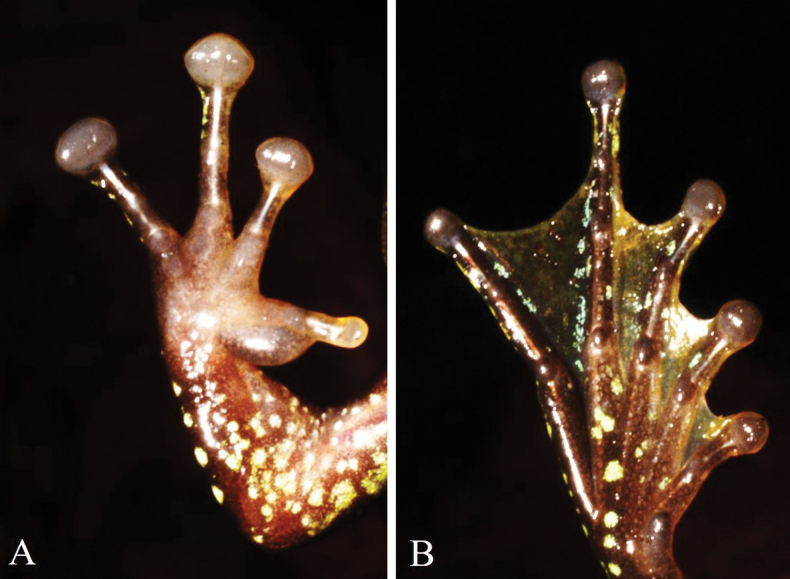
A. Palmar aspect of right hand; B. Plantar aspect of right foot of the male holotype (IEBR A. 5139) of *Amolops cuongi* sp. nov.

##### Variation.

Morphometric measurements of the type series are shown in Table 1 and representative photographs of paratypes and referred specimen in life are shown in Fig. 6. Specimens vary in body size and most dramatically in colouration in life (Fig. 6). Two paratypes (ILS H.3665) and ILS H.3666 (Fig. 6B) have diffuse-edged blotches of bluish grey on their dorsal surfaces. The dorsal surfaces of ILS H.3683 (Fig. 6C) are dark brown with diffuse-edged blotches of yellowish green and copper and more distinct barring on the lateral surfaces of the hindlimbs. The dorsal surfaces of ILS and ILS H.3663 (Fig. 6G) are pale green and copper, and the dorsal surfaces of the body and limbs in HLNP2017 1409 00030 (Fig. 6F) and ILS H.3662 (Fig. 6E) are almost entirely copper. The flanks of ILS H.3683, ILS H.3684, HLNP2017 1409 00030, ILS H.3662, and ILS H.3663 are pale green with some faint brown blotches. Interdigital webbing colouration does not differ between the upper and lower surfaces but is variable between specimens and is pale yellow in ILS H.3683, HLNP2017 1409 00030, ILS H.3662 and ILS H.3664 (versus greyish brown in other specimens in the series). The colouration of the ventral surface of head, throat, chest, and abdomen is also highly variable, creamy grey in ILS H.3666, pale green in HLNP2017 1409 00030, ILS H.3662, ILS H.3663, ILS H.3664, ILS H.3673, ILS H.3683, and ILS H.3684. Colouration of ventral surface of hindlimbs variable, pale brown with white granules on thigh in HLNP2017 1409 00030 and ILS H.3683, pale brown with small white and pale yellow blotches on ventral surface of thigh in ILS H.3662, pale brown in ILS H.3663, pale brown with dense greenish cream small blotches in ILS H.3664 and ILS H.3673, pale brown with dense greenish cream and bluish grey small blotches in ILS H.3664 and ILS H.3684; lip stripe colouration also variable, green in all individuals except holotype and ILS H.3666. Iris colour is variable, gold and metallic orange with black reticulations in ILS H.3683 and ILS H.3684, HLNP2017 1409 00030, ILS H.3662, ILS H.3663, ILS H.3664. In ILS H.3662, ILS H.3666, ILS H.3673; ventral surfaces of throat, chest, belly, and abdomen only weakly granular, small spinules not present on in ILS H.3666, ILS H.3673, ILS H.3675 but there are small white tubercles in their place. ILS H.3684 with very dense area of white spinules almost extending to axilla, these are also present on the axilla in ventral view; vomerine teeth are weakly developed in ILS H.3671, ILS H.3662, ILS H.3664, ILS H.3665, ILS H.3672, ILS H.3683, ILS H.3673 and ILS H.3674 and not present in HLNP2017 1409 00030, ILS H.3663, ILS H.3666, ILS H.3675, ILS H.3686, ILS H.3684, ILS H.3675 and ILS H.3670.

**Figure 5.**
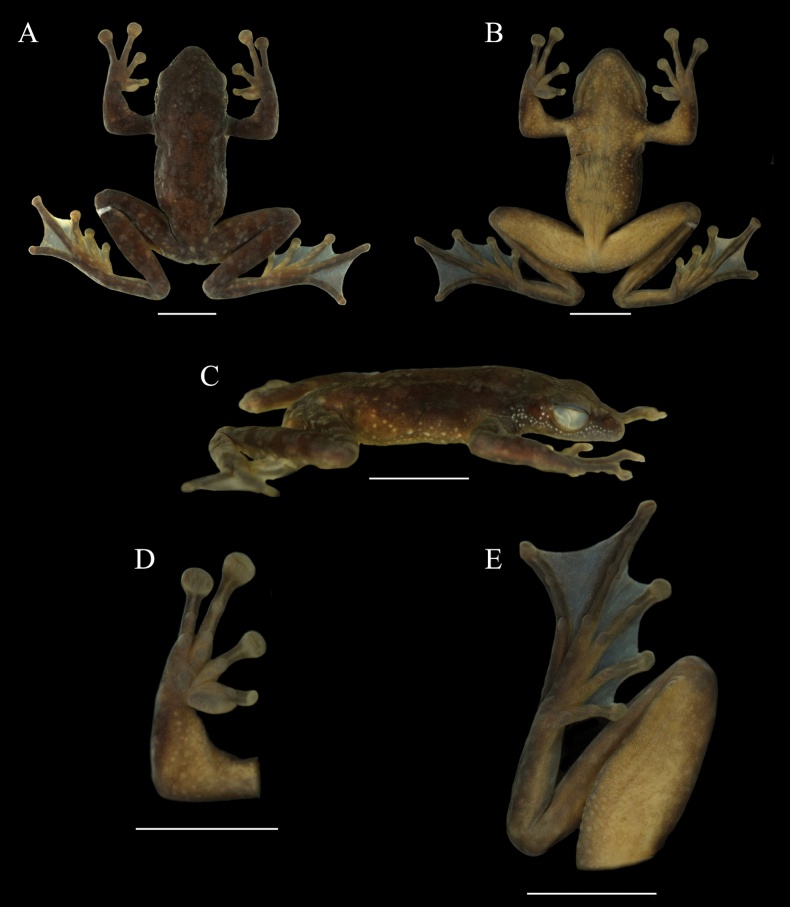
*Amolops cuongi* sp. nov. in preservative, adult male paratype ILS H.3665. A. Dorsal view; B. Ventral view; C. Lateral view; D. Palmar aspect of right hand; E. Plantar aspect of right foot. Scale bars 10 mm.

**Figure 6.**
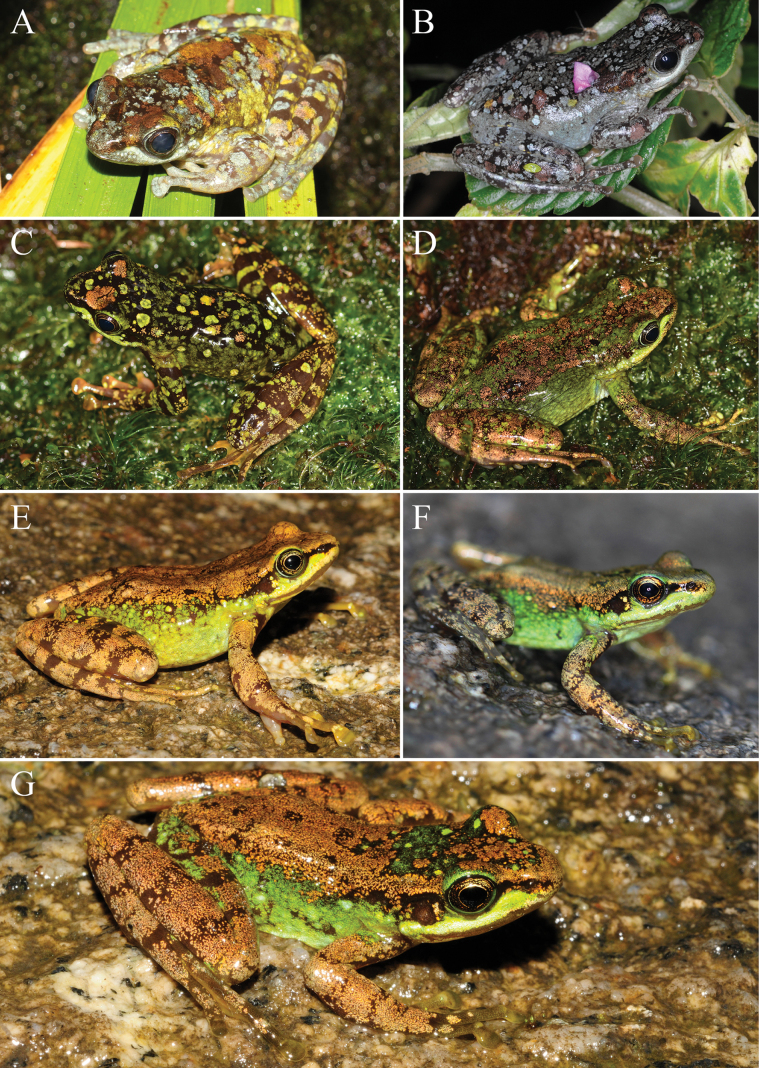
Dorsolateral view of *Amolops cuongi* sp. nov. in life. A. Adult male paratype ILS H.3665; B. Adult female paratype ILS H.3666; C. Adult male paratype ILS H.3683; D. Adult female paratype ILS H.3684; E. Adult female paratype ILS H.3662; F. Subadult paratype HLNP2017 1409 00030; G. Subadult paratype ILS H.3663.

**Table 1.** Measurements (in mm) and proportions of the type series of *Amolops cuongi* sp. nov. (for other abbreviations see Material and methods). SA = subadult; HT = holotype; PR = paratype; REF = Referred specimen.

	IEBR A.5139	ILS H.3665	ILS H.3667	ILS H.3683	ILS H.3674	ILS H.3685	ILS H.3686	ILS H.3670	IEBR A.5140	IEBR A.5141	ILS H.3666	ILS H.3668	ILS H.3664	ILS H.3672	ILS H.3684	HLNP2017 1409 00030	ILS H.3663	ILS H.3669	ILS H.3675
	LC2020.82	FAN 40	FAN 45	LNT 2983	LNT 3015	LNT 2986	LNT 2987	JJLR 05021	LC2020.179	LC2020.181	FAN 41	FAN 46	FAN 04	LNT 2971	LNT 2985	FAN 01	FAN 03	FAN 47	LNT 3018
	HT	PR	PR	PR	PR	PR	PR	PR	PR	PR	PR	PR	PR	PR	PR	REF	PR	PR	PR
	♂	♂	♂	♂	♂	♂	♂	♂	♀	♀	♀	♀	♀	♀	♀	SA	SA	SA	SA
SVL	36.0	36.0	35.5	36.8	36.9	34.8	33.9	35.6	39.1	37.9	41.7	44.4	44.1	39.5	41.5	22.5	27.0	25.1	28.4
HL	12.2	13.5	12.5	13.4	14.0	12.7	12.7	13.1	13.7	13.3	14.4	15.1	15.8	15.0	14.5	10.9	10.8	9.1	10.5
HW	11.8	11.5	11.3	11.9	12.3	11.9	11.7	11.8	13.0	13.1	13.7	14.2	14.2	13.7	13.9	9.8	9.5	8.1	9.5
SE	5.3	5.7	5.8	6.2	5.6	5.2	5.6	5.5	6.1	6.0	5.9	6.9	6.3	6.3	6.5	5.2	5.0	4.2	4.8
SND	3.0	2.9	2.6	3.2	2.8	2.4	2.6	2.6	3.4	3.2	2.7	3.4	3.0	2.6	2.7	2.6	2.6	2.2	1.7
END	2.3	2.7	3.2	2.9	2.8	2.9	3.0	2.9	2.7	2.8	3.1	3.5	3.4	3.7	3.8	2.6	2.4	2.0	3.1
IND	4.2	3.9	4.5	4.8	4.7	4.4	4.5	4.6	4.5	4.4	4.9	5.1	5.4	4.8	5.2	4.0	4.0	3.6	3.8
IOD	4.2	3.7	4.2	4.1	4.0	4.0	3.7	3.7	4.7	4.4	4.6	4.8	3.4	3.7	3.9	3.3	4.5	3.1	3.7
ED	5.0	4.7	4.4	4.7	4.5	4.9	4.6	4.6	5.5	5.4	4.9	4.9	5.5	5.7	5.1	4.0	4.1	3.5	4.1
TD	1.5	1.2	1.2	1.5	1.4	1.2	1.7	1.7	1.5	1.4	1.3	1.7	1.8	1.6	1.5	1.2	1.6	1.2	1.2
TED	1.6	1.1	1.6	1.3	1.2	1.0	1.4	1.7	2.0	1.8	1.3	1.9	1.9	1.6	1.8	1.0	1.2	1.2	1.0
HND	12.2	12.1	19.2	12.0	11.3	11.3	14.1	11.2	13.9	13.6	14.1	15.2	15.6	14.1	14.4	9.4	8.3	12.7	9.5
FTD	2.5	2.6	2.3	2.2	2.2	2.5	2.3	2.0	2.7	2.5	2.5	2.9	3.7	2.7	3.0	1.4	1.7	1.3	1.7
FL	17.9	19.3	19.4	17.9	19.5	17.2	19.0	19.2	20.0	19.3	21.0	21.8	22.3	20.9	20.3	15.1	15.7	13.3	14.6
TL	20.2	20.4	19.5	19.6	19.8	19.2	19.9	19.2	22.5	22.4	22.1	23.4	24.0	22.3	21.6	16.8	17.5	14.6	16.4
FOT	19.0	19.1	20.9	20.8	19.5	19.4	18.7	19.6	22.0	21.2	2.6	22.2	23.9	21.9	22.3	14.9	15.5	12.7	15.2
HTD	1.6	1.7	1.8	1.3	1.3	1.7	1.7	1.6	1.6	1.5	1.8	2.2	2.4	1.8	2.0	1.0	1.5	1.0	1.0
HL/SVL	0.34	0.38	0.35	0.36	0.38	0.36	0.37	0.37	0.35	0.35	0.35	0.34	0.36	0.38	0.35	0.48	0.40	0.36	0.37
HW/SVL	0.33	0.32	0.32	0.32	0.33	0.34	0.35	0.33	0.33	0.35	0.33	0.32	0.32	0.35	0.33	0.44	0.35	0.32	0.33
SE/SVL	0.15	0.16	0.16	0.17	0.15	0.15	0.17	0.15	0.16	0.16	0.14	0.16	0.14	0.16	0.16	0.23	0.19	0.17	0.17
ED/HL	0.41	0.35	0.35	0.35	0.32	0.39	0.36	0.35	0.40	0.41	0.34	0.32	0.35	0.38	0.35	0.37	0.38	0.38	0.39
ED/SE	0.94	0.82	0.76	0.76	0.80	0.94	0.82	0.84	0.90	0.90	0.83	0.71	0.87	0.90	0.78	0.77	0.82	0.83	0.85
TD/ED	0.30	0.26	0.27	0.32	0.31	0.24	0.37	0.37	0.27	0.26	0.27	0.35	0.33	0.28	0.29	0.30	0.39	0.34	0.29
FLL/SVL	0.34	0.34	0.54	0.33	0.31	0.32	0.42	0.31	0.36	0.36	0.34	0.34	0.35	0.36	0.35	0.42	0.31	0.51	0.33
FTD/ED	0.50	0.55	0.52	0.47	0.49	0.51	0.50	0.43	0.49	0.46	0.51	0.59	0.67	0.47	0.59	0.35	0.41	0.37	0.41
FTD/SVL	0.07	0.07	0.06	0.06	0.06	0.07	0.07	0.06	0.07	0.07	0.06	0.07	0.08	0.07	0.07	0.06	0.06	0.05	0.06
TD/FTD	0.60	0.46	0.52	0.68	0.64	0.48	0.74	0.85	0.56	0.56	0.52	0.59	0.49	0.59	0.50	0.86	0.94	0.92	0.71

##### Distribution.

*Amolops cuongi* sp. nov. is currently known only from the Hoang Lien Range in northern Vietnam, including Tam Duong, Lai Chau Province and Sa Pa and Bat Xat, Lao Cai Province (Fig. 1).

##### Etymology.

The new species is named after Dr. Cuong The Pham, our colleague from Institute of Biology, Vietnam to honour his contributions to the herpetological research in Vietnam, particularly in the taxonomy of the anuran species complexes. For the common names we suggest Cuong’s Torrent Frog (English) and Ếch bám đá cường (Vietnamese).

##### Ecological notes.

*Amolops cuongi* sp. nov. is recorded from both disturbed and primary evergreen and bamboo forest with a relatively closed canopy (Suppl. material 2: fig. S1). Nearly all individuals have been found at night, perched on streamside vegetation ≤2.5 m from the ground alongside clear, fast-moving water, along ca 3.0 m wide streams or waterfalls. The holotype was collected from a small stream with large mossy rocks in subtropical forest consisting of medium and large hardwood trees and shrubs. The air temperature when specimens were collected was 17–25 °C and relative humidity was 80–90%. Calls were not heard in any of the survey work. The breeding season is likely to encompass September and October and possibly longer as all males were observed with velvety nuptial pads lacking spines in both September and October and four of the six females collected in September were gravid. The single female collected in June was not gravid. The tadpole and breeding behaviour of this species is unknown. Other amphibian species found at the collection site in Ho Thau Commune were *Bombina microdeladigitora* Liu, Hu, Yang, *Oreolalax sterlingae* Nguyen, Phung, Le, Ziegler & Böhme, *Leptobrachella* sp., *Leptobrachium ailaonicum* (Yang, Chen & Ma), *Nanorana yunnanensis* (Anderson), and *Atympanophrys gigantica* (Liu, Hu & Yang) on Mount Pu Ta Leng, *Amolops minutus* and *Amolops spicalinea* Nguyen, Tapley, La & Rowley on Mount Fansipan. On Mount Nam Kang Ho Tao this species is sympatric with *A. minutus* and *Amolops daorum*.

##### Conservation status.

This new species is currently known from between 1928–2784 m a.s.l. at three localities in Lao Cai and Lai Chau provinces, up to 48 km apart in the Hoang Lien Range (Mount Pu Ta Leng, Mount Fansipan and Mount Nam Kang Ho Tao). It was not detected in over a dozen field surveys along the Cat Cat River in Hoang Lien National Park (22.3214°N 103.8264°E, 1244 m a.s.l.) in evergreen forest indicating that it may not occur at lower elevation. The species’ estimated range is a narrow band of high-elevation habitat along the boundary of Lao Cai and Lai Chau provinces in Vietnam and into Yunnan Province in China. Its EOO is currently predicted to 2281 km^2^ (Fig. 1). It occurs in a single threat defined location. The habitat of this species in all three localities is degraded, at elevations below 2000 m a.s.l., forest is being degraded in riparian habitat to establish cardamom plantations. On Mount Fansipan, the forest in which this species occurs is being negatively impacted by fuelwood collection for the tourism industry. A tourist camp is located just 500 m from the streams where this species was recorded on Mount Fansipan and effluent from the tourist camps is discharged into the stream. The habitat in higher elevations on Mount Fansipan is polluted with litter discarded by tourists and the gravel stream substrate has been intensively mined to line trekking routes, and this may have an impact on oviposition sites and larval development sites (see Nguyen et al. 2020 for details). Northern Vietnam is increasingly impacted by forest fires (Le et al. 2014; Vadrevu et al. 2019) and forest fires also threaten the habitat of *Amolops cuongi* at high elevation sites with natural forest have a greater probability of fire in northern Vietnam (Trang et al. 2022). Climate change is also a threat to the new species and a warmer climate could lead to a net loss in range (e.g., Duan et al. 2016). The fungal pathogen, *Batrachochytrium dendrobatidis*, was not detected from 30 samples collected from four different anuran amphibian species at approximately 2650 m a.s.l. on Mount Fansipan in September 2017; including four samples from *Amolops cuongi*, it was also not detected from 20 samples from eight different anuran amphibian species at approximately 2000 m a.s.l. on Mount Fansipan in June 2018, including four samples from *Amolops cuongi* (Tapley et al. 2020; reported as *Amolops* sp. 1). Given the relatively small range, number of locations and ongoing habitat loss and modification, this species qualifies for being assessed as Vulnerable in accordance with the IUCN Red List of Threatened Species categories and criteria B1ab(iii) (see IUCN 2012).

##### Comparisons.

As molecular and morphological analyses confirm that the new species belongs to the *A. mantzorum* group, we compare the new species with other members of the *A. mantzorum* group: *Amolops cuongi* sp. nov. differs from *A. ailao* by the absence of a glandular dorsolateral fold (vs present in *A. ailao*); a smaller ratio of TD/ED (TD/ED 0.24–0.36 vs 0.52 in males; 0.26–0.35 vs 0.58 in females in *A. ailao*); tibiotarsal articulation reaching to the nostril (vs tibiotarsal articulation reaching beyond anterior corner of eye in *A. ailao*); the absence of brown mottling on the throat (vs present in *A. ailao*); and granular ventral surface (vs smooth in *A. ailao*) (Tang et al. 2023); from *A. dafangensis* by having a smaller size in males (SVL 33.9–36.9 mm vs 43.2–46.8 mm in *A. dafangensis*), glandular dorsolateral folds absent (vs present in *A. dafangensis*), and the absence of brown mottling on the throat (vs present in *A. dafangensis*) (Li et al. 2024); from *A. granulosus* by having a smaller size in females (SVL 37.9–44.4 mm vs 51.9 mm in *A. granulosus*), tympanum visible (vs invisible in *A. granulosus*), and having a dorsal body colouration of dark brown with diffuse-edged blotches of bluish grey, copper and yellowish green or pale green and copper (vs brown with black spots in *A. granulosus*) (Liu and Hu 1961; Fei et al. 2009, 2010); from *A. jinjiangensis* by having a smaller size (SVL 33.9–36.9 mm vs 43.0–52.0 mm in males; 37.9–44.4 mm vs 58.0–65.0 mm in females in *A. jinjiangensis*), tympanum visible (vs invisible in *A. jinjiangensis*), and glandular dorsolateral folds absent (vs present in *A. jinjiangensis*), and the absence of small granules on the tympanum (vs present in *A. jinjiangensis*) (Yang et al. 1983; Yang 2008; Fei et al. 2009); from *A. lifanensis* by having a smaller size (SVL 33.9–36.9 mm vs 52.0–56.0 mm in males; 37.9–44.4 mm vs 61.0–79.0 mm in females in *A. lifanensis*), and tympanum distinct (vs invisible in *A. lifanensis*) (Su et al. 1986; Fei et al. 2009); from *A. loloensis* by having a smaller size (SVL 33.9–36.9 mm vs 54.5–62.0 mm in males; 37.9–44.4 mm vs 69.5–77.5 mm in females in *A. loloensis*), tympanum distinct (vs invisible in *A. loloensis*), dorsal colour pattern (dark brown with diffuse-edged blotches of bluish grey, copper, and yellowish green or pale green and copper vs dark green with many large brown spots in *A. loloensis*), tibiotarsal articulation reaching to the nostril (vs tibiotarsal articulation reaching to the eye in *A. loloensis*), head longer than wide (vs wider than long or long as wide in *A. loloensis*), and relative finger lengths: I<II<IV<III (vs I = II<IV<III in *A. loloensis*) (Liu 1950; Fei et al. 2009); from *A. mantzorum* by having a smaller size (SVL 33.9–36.9 mm vs 48.8–57.0 mm in males, 37.9–44.4 mm vs 57.5–72.0 mm in females in *A. mantzorum*), tympanum distinct (vs invisible in *A. mantzorum*), and hind limbs with dark crossbars (vs without dark crossbars in *A. mantzorum*) (Liu 1950; Fei et al. 2009, 2017); from *A. minutus* by having a smaller tympanum (TD/ED 0.24–0.37 vs 0.38–0.41 in males; 0.26–0.35 vs 0.38–0.40 in females in *A. minutus*), a larger disc of finger III (FTD/ED 0.43–0.52 vs 0.29–0.33 in males, 0.46–0.67 vs 0.36–0.39 in females in *A. minutus*; FTD/SVL 0.07–0.08 vs 0.04–0.05 in *A. minutus*), a granular ventral surfaces and flanks (vs smooth in *A. minutus*), the presence of a band of small spinules running from below nares, along upper lip, around lower half of eye, between tympanum and eye and rear axis of mandibles (vs spinules typically only present posterior of tympanum in *A. minutus*), and the absence of dorsolateral glandular folds (vs present in *A. minutus*) (Orlov et al. 2007; Pham et al. 2019; Suppl. material 2: fig. S2); from *A. sangzhiensis* by having a smaller size (SVL 33.9–36.9 mm vs 40.3–40.9 mm in males, 37.9–44.4 mm vs 52.6–57.7 mm in females in *A. sangzhiensis*), and tympanum smooth (vs covered in fine granules in *A. sangzhiensis*) (Qian et al. 2023); from *A. shuichengicus* by having a smaller size in females (SVL 37.9–44.4 mm vs 48.5–55.5 mm in *A. shuichengicus*), the absence of dorsolateral glandular folds (vs present in *A. shuichengnicus*), and the absence of a cream maxillary gland (vs present in *A. shuichengnicus*) (Lyu et al. 2019); and from *A. tuberodepressus* by having a smaller size (SVL 33.9–36.9 mm vs 44.0–57.0 mm in males; SVL 37.9–44.4 mm vs 61.0–70 mm in *A. tuberodepressus* in females), tibiotarsal articulation reached to the nostril (vs tibiotarsal articulation reaching beyond tip of snout in *A. tuberodepressus*), tympanum distinct (vs invisible in *A. tuberodepressus*), and different dorsal colour pattern (green with some dark or pale green spots and irregular small black dots in *A. tuberodepressus*) (Liu et al. 2000; Fei et al. 2009).

## ﻿Discussion

Our new finding brings the total number of *Amolops* species from Hoang Lien Mountain Range to eight, namely *A. cuongi*, *A. cucae* (Bain, Stuart & Orlov), *A. daorum* (Bain, Lathrop, Murphy, Orlov & Ho), *A. mengyangensis* Wu & Tian, *A. minutus* Orlov & Ho, *A. spicalinea*, *A. shihaitaoi* Wang, Li, Du, Hou & Yu, and *A. viridimaculatus* Jiang (Frost 2025). Of which, *A. cucae*, *A. daorum*, *A. mengyangensis*, and *A. spicalinea* are placed in the *A. monticola* group, *A. shihaitaoi* within the *A. ricketti* group, *A. viridimaculatus* within the *A. viridimaculatus* group (Wu et al. 2020; Patel et al. 2021), and *A. cuongi* and *A. minutus* within the *A. mantzorum* group. *Amolops minutus* is currently known from Bat Xat, Sa Pa, and Tam Duong districts, Lai Chau Province, and Muong La District, Son La Province (Orlov and Ho 2007; Pham et al. 2019; Liu et al. 2024; this study). In terms of genetic distance, *Amolops cuongi* is closely related to *A. shuichengicus* based on the 16S rRNA gene, *A. minutus* according to the ND2 gene, and *A. loloensis* in terms of the cyt*b* gene, but the new species differs from a minimum of 1.35% (*A. shuichengicus* according to the 16S rRNA gene) to a maximum of 19.14% (*A. lifanensis* in the ND2 gene).

Phylogenetically, the new species is the most distinct taxon within the *A. mantzorum* group, as it is placed in a clade sister to all remaining group members, except for *A. lifanensis*. The latter is weakly recovered as a member of the *A. mantzorum* group with insignificant support values (BPP = 0.66, UFB = 86). Similarly, previous molecular studies demonstrate that *A. lifanensis* is either paraphyletic to the *A. mantzorum* group (Wiens et al. 2009; Wu et al. 2020) or clusters with the group with a low support value (Qian et al. 2023). The phylogenetic placement of *A. lifanensis* should be investigated in future studies using multilocus data, including both nuclear and mitochondrial markers.

*Amolops cuongi* and *A. minutus* were found in sympatry in the evergreen forest of Lai Chau Province. *Amolops cuongi* could occur more widely and potentially at lower elevations. Establishing the true lower elevation range of this species should also be considered as it may inform future survey effort and extinction risk assessments. Future research should aim to elucidate more life history information including the identification of breeding habitat and formal scientific description of the call and larvae.

The description of *Amolops cuongi* further highlights the significance of the Hoang Lien Range as an area of exceptional amphibian species diversity. Ten amphibians have been described from the Hoang Lien Range as new species to science within the last decade (Matsui et al. 2017; Tapley et al. 2017, 2018, 2020; Kropachev et al. 2019; Nguyen et al. 2021, 2024a, b, 2025). With the exception of *Rhacophorus duboisi* Ohler, Marquis, Swan & Grosjean, 2000, the entire amphibian assemblage above 2600 m a.s.l. on Mount Fansipan consists of species that were described as new species to science since 2013 (Nguyen et al. 2013; Rowley et al. 2013; Tapley et al. 2018; this study) and all of these newly described species are highly threatened (IUCN SSC Amphibian Specialist Group 2021a, b, c) or likely qualify for being assessed as Vulnerable on the basis of available information (this study). The recent scientific discovery of *Oreolalax adelphos* Nguyen Tapley, Kane, Tran, Cui & Rowley on the summit of Mount Po Ma Lung indicates that further species, or even assemblages, on other mountain summits in the Hoang Lien Range may await formal scientific description. Further surveys and integrative taxonomic work should be undertaken soon as these areas are increasingly degraded by infrastructural developments and habitat degradation associated with tourism. Furthermore, climate change is highly likely to be particularly problematic for these high elevation, range restricted species. Several studies have shown that some amphibian species will migrate to higher elevations as the climate warms (Duan et al. 2016; Sillero 2021); for species already occurring at high elevation, this is simply not possible and the ranges of these already range-restricted species can only contract further. This underscores the significance of protecting the last remaining tracts of high elevation natural forest in Vietnam as climatic refuges for amphibians.

## Supplementary Material

XML Treatment for Amolops cuongi

## ﻿Additional information

### Use of AI

No use of AI was reported.

### Author ORCIDs

Anh Van Pham https://orcid.org/0000-0002-6023-3418

Chung Van Hoang https://orcid.org/0000-0002-0709-974X

Benjamin Tapley https://orcid.org/0000-0002-9787-3793

Luan Thanh Nguyen https://orcid.org/0000-0002-4663-125X

Thomas Ziegler https://orcid.org/0000-0002-4797-609X

Jodi J. L. Rowley https://orcid.org/0000-0002-2011-9143

Truong Quang Nguyen https://orcid.org/0000-0002-6601-0880

Minh Duc Le https://orcid.org/0000-0002-2953-2815

### Data availability

All of the data that support the findings of this study are available in the main text or Supplementary Information.

## Supplementary materials

Supplementary material 1 Supplementary tables Data type docx Explanation note **table S1.** Samples used for the phylogenetic analyses in this study. **table S2.** Uncorrected pairwise genetic distance (%) between members of the genus *Amolops* estimated from 16S sequences. **table S3.** Uncorrected pairwise genetic distance (%) between members of the genus *Amolops* estimated from ND2 sequences. **table S4.** Uncorrected pairwise genetic distance (%) between members of the genus *Amolops* estimated from cytb sequences https://binary.pensoft.net/file/1445740This dataset is made available under the Open Database License (http://opendatacommons.org/licenses/odbl/1.0/). The Open Database License (ODbL) is a license agreement intended to allow users to freely share, modify, and use this Dataset while maintaining this same freedom for others, provided that the original source and author(s) are credited.Anh Van Pham, Chung Van Hoang, Benjamin Tapley, Luan Thanh Nguyen, Hanh Huu Nguyen, Toi Van La, Thomas Ziegler, Jodi J. L. Rowley, Truong Quang Nguyen, Minh Duc Le

Supplementary material 2 Supplementary figures Data type docx Explanation note **fig. S1.** Habitat of *Amolops cuongi* sp. nov. in the Hoang Lien Range. **fig. S2.** (A–C) *Amolops cuongi* sp. nov. adult male paratype ILS H.3665 dorsal, ventral and lateral views in preservative. https://binary.pensoft.net/file/1445741This dataset is made available under the Open Database License (http://opendatacommons.org/licenses/odbl/1.0/). The Open Database License (ODbL) is a license agreement intended to allow users to freely share, modify, and use this Dataset while maintaining this same freedom for others, provided that the original source and author(s) are credited.Anh Van Pham, Chung Van Hoang, Benjamin Tapley, Luan Thanh Nguyen, Hanh Huu Nguyen, Toi Van La, Thomas Ziegler, Jodi J. L. Rowley, Truong Quang Nguyen, Minh Duc Le
