# Supplementary material for: ﻿Amolops cuongi (Amphibia, Anura, Ranidae), a new species from the Hoang Lien Range, Vietnam
Source: Zookeys. 2025 Oct 22;1256:235–57. doi: 10.3897/zookeys.1256.158846 (PMC12572962; doi:10.3897/zookeys.1256.158846)
Supplement: Supplementary material 1 — Supplementary tables [file zookeys-1256-235_article-158846__-s001.docx]

***Amolops cuongi*** (Amphibia: Anura: Ranidae)**, a new species** from the Hoang Lien Range, Vietnam

Supplementary table 1, 2, 3, 4

**Table 1.** Samples used for the phylogenetic analyses in this study; the GenBank accession numbers generated in this study are highlighted in bold.

|  | **Species** | **Voucher** | **Locality** | **16S** | **ND2** | **cyt*b*** |
| --- | --- | --- | --- | --- | --- | --- |
| **1** | *Amolops cuongi* sp. nov. | IEBR A.5140 | Vietnam: Tam Duong, Lai Chau | **PX113529** | **PX119670** | **PX119668** |
| **2** | *Amolops cuongi* sp. nov. | IEBR A.5141 | Vietnam: Tam Duong, Lai Chau | **PX113530** | **PX119671** | **PX119669** |
| **3** | *Amolops cuongi* sp. nov. | IEBR A.5139 | Vietnam: Tam Duong, Lai Chau | / | **PX119672** | **/** |
| **4** | *Amolops cuongi* sp. nov. | ILS H.3665 | Vietnam: Tam Duong, Lai Chau | **PX114202** | **/** | **PX121563** |
| **5** | *Amolops cuongi* sp. nov. | ILS H.3669 | Vietnam: Tam Duong, Lai Chau | **PX113534** | **/** | **PX121565** |
| **6** | *Amolops cuongi* sp. nov. | ILS H.3667 | Vietnam: Tam Duong, Lai Chau | **PX113527** | **/** | **PX121564** |
| **7** | *Amolops ailao* | GXNU YU000001 | Mt. Ailao, Xinping, Yunnan, China | MN650751 | / | MN650743 |
| **8** | *Amolops dafangensis* | MT DF20230601002 | China: Dafang, Guizhou | OR936315 | / | / |
| **9** | *Amolops granulosus* | 20130258 | China: Hongya, Sichuan | MH922934 | MH922934 | MH922934 |
| **10** | *Amolops granulosus* | SYS a005315 | China: Hongya, Sichuan | MK604850 | / | / |
| **11** | *Amolops granulosus* | / | Anxian, Sinchuan, China | / | / | KJ008444 |
| **12** | *Amolops granulosus* | / | Anxian, Sinchuan, China | / | / | KJ008439 |
| **13** | *Amolops jinjiangensis* | SCUM 050435CHX | China: Deqing, Yunnan | EF453741 | / | / |
| **14** | *Amolops jinjiangensis* | CIB-XM6120 | Benzilan Town of Deqin County, Yunnan Province inChina | MZ292455 | MZ292455 | MZ292455 |
| **15** | *Amolops* cf. *mantzorum* | / | Zhongdian, Yunnan, China | / | / | KJ008372 |
| **16** | *Amolops* cf. *mantzorum* | / | Zhongdian, Yunnan, China | / | / | KJ008373 |
| **17** | *Amolops lifanensis* | SCUM045801HX | Maoxian, Sichuan, China | MN953702 | MN958760 | / |
| **18** | *Amolops lifanensis* | SCUM045803HX | Maoxian, Sichuan, China | MN953703 | MN958761 | / |
| **19** | *Amolops loloensis* | SYS a005351 | China: Zhaojue, Sichuan | MK573806 | / | / |
| **20** | *Amolops loloensis* | / | Huanyuan, Sichuan, China | / | / | KJ008430 |
| **21** | *Amolops loloensis* | / | Qiliba, Zhaojue, Sichuan, China | / | / | KJ008427 |
| **22** | *Amolops loloensis* | SM-ZDTW-01 | Shimian County, Sichuan, China | KT750963 | KT750963 | KT750963 |
| **24** | *Amolops mantzorum* | SYS a005366 | China: Baoxing, Sichuan | MK604862 | / | / |
| **24** | *Amolops mantzorum* | / | Xiling Snow Mountain, Dayi County, Sichuan Province, China | KJ546429 | KJ546429 | KJ546429 |
| **25** | *Amolops mantzorum* | / | Wanba, Jiulong, Sichuan, China | / | / | KJ008297 |
| **26** | *Amolops mantzorum* | / | Wenxian, Gansu, China | / | / | KJ008339 |
| **27** | *Amolops mantzorum* ssp. | / | / | OL495260 | OL495260 | OL495260 |
| **28** | *Amolops mantzorum* ssp. | / | Maoxian, Sichuan, China | / | / | KJ008360 |
| **29** | *Amolops mantzorum* ssp. | / | Wanba, Jiulong, Sichuan, China | / | / | KJ008277 |
| **30** | *Amolops mantzorum xinduqiao* | / | Xinduqian, Sichuan, China | / | / | KJ008410 |
| **31** | *Amolops mantzorum xinduqiao* | / | Xinduqian, Sichuan, China | / | / | KJ008423 |
| **32** | *Amolops minutus* | IEBR A.5142 | Vietnam: Tam Duong, Lai Chau | PQ346023 | TDLC60 | MK941135 |
| **33** | *Amolops minutus* | IEBR A.6300 | Vietnam: Tam Duong, Lai Chau | PQ346024 | TDLC83 | / |
| **34** | *Amolops minutus* | IEBR 4342 (Holotype of A. ottorum) | Vietnam: Muong La, Son La | PQ346025 | / | / |
| **35** | *Amolops minutus* | TBU 06 | Vietnam: Muong La, Son La | / | / | MK941136 |
| **36** | *Amolops minutus* | KIZ 2023064 | China: Yuanyang, Yunnan | PQ346027 | / | / |
| **37** | *Amolops sangzhiensis* | CSUFT 901 | China: Sangzhi, Hunan | OQ079538 | / | / |
| **38** | *Amolops shuichengicus* | SYS a004956 | China: Shuicheng, Guizhou | MK604845 | / | / |
| **39** | *Amolops tuberodepressus* | SCUM050433CHX | China: Jingdong, Yunnan | MN953729 | MN958786 | / |
| **40** | *Amolops tuberodepressus* | YU20160272 | China: Xinping, Yunnan | MN650757 | / | / |
| **41** | *Amolops tuberodepressus* | / | Mt. Wuliang, Jingdong, Yunnan, China | / | / | KJ008426 |
| **42** | *Amolops adicola* | BNHS 6121 | India: Arunachal Pradesh | MZ229772 | MZ231116 | / |
| **43** | *Amolops akhaorum* | FMNH 271355 | Laos: Luang Namtha | FJ417158 | FJ417207 | / |
| **44** | *Amolops aniqiaoensis* | SYNU 04II6015 | China: Medog, Tibet | MN953655 | MN958714 | / |
| **45** | *Amolops archotaphus* | FMNH 271708 | Thailand: Chiang Mai | MN953659 | MN958718 | / |
| **46** | *Amolops beibengensis* | KIZ 016397 | Medog, Xizang, China | MN953662 | MN958721 | / |
| **47** | *Amolops* cf. *bellulus* | CAS 233991 | China: Tengchong, Yunnan | FJ417127 | FJ417176 | / |
| **48** | *Amolops chaochin* | SCUM045818HX | China: Anxian, Sichuan | MN953669 | FJ417179 | / |
| **49** | *Amolops cucae* | AMNH 168727 | Vietnam: Van Ban, Lao Cai | FJ417144 | FJ417193 | / |
| **50** | *Amolops compotrix* | FMNH 256500 | Laos: Nakai, Khammouane | FJ417141 | FJ417190 | / |
| **51** | *Amolops chunganensis* | KIZYPX18652 | China: Chengkou, Chongqing | MN953670 | MN958728 | / |
| **52** | *Amolops cremnobatus* | KIZ 011622 | Puhu National Reserve, Thanh Hoa, Vietnam | MN953673 | MN958731 | / |
| **53** | *Amolops daorum* | ROM 38503 | Vietnam: Sa Pa, Lao Cai | FJ417151 | FJ417200 | / |
| **54** | *Amolops* cf. *daorum* | FMNH 255353 | Laos: Vieng Tong, Houaphan | FJ417147 | FJ417196 | / |
| **55** | *Amolops deng* | KIZ 14116 | China: Zayü, Tibet | MN953695 | MN958752 | / |
| **56** | *Amolops iriodes* | AMNH 163926 | Vietnam: Vi Xuyen, Ha Giang | FJ417152 | FJ417201 | / |
| **57** | *Amolops formosus* | KIZ 012533 | Gyirong, Xizang, China | MN953682 | MN958739 | / |
| **58** | *Amolops himalayanus* | KIZ 040227 | Mabu, Ilam, Nepal | MN953713 | MN958771 | / |
| **59** | *Amolops kaulbacki* | SCUM050403CHX | Pianma, Yunnan, China | MN953737 | MN958794 | / |
| **60** | *Amolops kohimaensis* | WIIADA 751 | India: Nagaland | MZ229774 | MZ231118 | / |
| **61** | *Amolops marmoratus* | FMNH 271696 | Wachiratarn Falls, Amphoe Chom Thong, Chiangmai, Thailand | MN953709 | MN958767 | / |
| **62** | *Amolops mengdingensis* | KIZ 20160266 | China: Mengding, Yunnan | MK501809 | MK501815 | / |
| **63** | *Amolops medogensis* | SYNU 04II6216 | Medog, Xizang, China | MN953710 | MN958768 | / |
| **64** | *Amolops monticola* | WIIADA 544 | India: South Sikkim | MZ229773 | MZ231117 | / |
| **65** | *Amolops nyingchiensis* | KIZ 16415 | China: Medog, Tibet | MN953718 | MN958776 | / |
| **66** | *Amolops pallasitatus* | SYNU 1507034 | Dinggyê, Xizang, China | MK573816 | / | / |
| **67** | *Amolops panhai* | FMNH 268355 | Huay Yang National Park, Prachuap Khiri Khan, Thailand | MN953720 | MN958778 | / |
| **68** | *Amolops putaoensis* | GXNU W011 | Myanmar: Putao, Kachin | MT901383 | MT901213 | / |
| **69** | *Amolops spinapectoralis* | KIZ 010110 | Phong Dien District, Thua Thien Hue, Vietnam | MN953769 | MN958826 | / |
| **70** | *Amolops truongi* | ZVNU.2022.01 | Vietnam: Muong La, Son La | / | OP157200 | / |
| **71** | *Amolops tuanjieensis* | GXNU YU110003 | China: Tuanjie, Yunnan | MN832772 | MN832756 | / |
| **72** | *Amolops viridimaculatus* | SYS a003813 | China: Mt. Gaoligong, Yunnan | MK604836 | / | / |
| **73** | *Amolops viridimaculatus* | KIZ 48487 | China: Tengchong, Yunnan | MN953731 | MN958788 | / |
| **74** | *Amolops viridimaculatus* | VNMN 010923 | Bat Xat, Lao Cai, Vietnam | MZ484725 | / | / |
| **75** | *Amolops vitreus* | FMNH 258183 | Laos: Phongsaly, Phongsaly | / | FJ417212 | / |
| **76** | *Amolops wangyufani* | KIZ 014067 | Zayü, Xizang, China | MN953740 | MN958796 | / |
| **77** | *Amolops wenshanensis* | KIZ 21425 | China: Xichou, Yunnan | MN953724 | MG996763 | / |
| **78** | *Amolops wuyiensis* | HDSK 0042 | Wuyishan, Fujian, China | MN953742 | MN958798 | / |
| **79** | *Amolops yangi* | KIZ 038645 | Lushui, Yunnan, China | PP097201 | PP102249 | / |
| **Outgroup** |  |  |  |  |  |  |
|  |  |  |  |  |  |  |
| **80** | *Odorrana jingdongensis* | KIZ046977 | China: Jingdong, Yunnan | MN953755 | MN958811 | / |

**Table 2. Uncorrected pairwise genetic distance (%) between members of the genus *Amolops* estimated from 16S sequences**

|  | **Species** | **1** | **2** | **3** | **4** | **5** | **6** | **7** | **8** | **9** | **10** | **11** | **12** |
| --- | --- | --- | --- | --- | --- | --- | --- | --- | --- | --- | --- | --- | --- |
| **1** | *Amolops cuongi* sp. nov. | **0.00–0.02** |  |  |  |  |  |  |  |  |  |  |  |
| **2** | *A. tuberodepressus* | 2.33–2.50 | **0.00** |  |  |  |  |  |  |  |  |  |  |
| **3** | *A. granulosus* | 1.74–2.11 | 1.72–2.11 | **0.00–0.19** |  |  |  |  |  |  |  |  |  |
| **4** | *A. mantzorum* | 2.53–3.10 | 1.92–2.50 | 2.11–2.69 | **0.00–0.57** |  |  |  |  |  |  |  |  |
| **5** | *A. shuichengicus* | 1.35–1.53 | 1.72 | 1.71–1.91 | 1.91–2.30 | **0.00** |  |  |  |  |  |  |  |
| **6** | *A. loloensis* | 1.73–1.91 | 1.72 | 1.71–1.91 | 1.91–2.30 | 1.14 | **0.00** |  |  |  |  |  |  |
| **7** | *A. minutus* | 1.93–2.31 | 1.72–2.30 | 1.72–2.49 | 1.53–2.50 | 1.33–1.72 | 1.33–2.10 | **0.00–0.57** |  |  |  |  |  |
| **8** | *A. ailao* | 2.05–2.51 | 1.92 | 1.91–2.11 | 1.72–2.11 | 1.72 | 1.72 | 0.57–1.14 | **0.00** |  |  |  |  |
| **9** | *A. jinjiangensis* | 1.73–1.91 | 1.33 | 1.33–1.52 | 1.52–1.91 | 1.14 | 0.76 | 1.14–1.71 | 1.33 | **0.00** |  |  |  |
| **10** | *A. dafangensis* | 2.32 | 2.31–2.32 | 1.84–2.08 | 2.08–2.55 | 1.84 | 1.61 | 1.84–2.31 | 1.61 | 1.15 | **0.00** |  |  |
| **11** | *A. sangzhiensis* | 1.54–1.72 | 1.14 | 1.14–1.33 | 1.33–1.72 | 0.95 | 0.57 | 0.95–1.52 | 1.14 | 0.19 | 0.92 | **0.00** |  |
| **12** | *A. lifanensis* | 5.14 | 6.14–6.56 | 5.10–5.31 | 6.14–6.56 | 5.72 | 5.72 | 6.34–6.55 | 6.57 | 6.12 | 5.44 | 5.92 | **0.00** |

**Table 3. Uncorrected pairwise genetic distance (%) between members of the genus *Amolops* estimated from ND2 sequences**

|  | **Species** | **1** | **2** | **3** | **4** | **5** | **6** | **7** | **8** |
| --- | --- | --- | --- | --- | --- | --- | --- | --- | --- |
| **1** | *Amolops cuongi* sp. nov. | **0.00–1.44** |  |  |  |  |  |  |  |
| **2** | *A. granulosus* | 9.49–9.96 | **0.00** |  |  |  |  |  |  |
| **3** | *A. tuberodepressus* | 10.66–11.32 | 6.89 | **0.00** |  |  |  |  |  |
| **4** | *A. jinjiangensis* | 9.24–9.94 | 6.32 | 5.26 | **0.00** |  |  |  |  |
| **5** | *A. mantzorum* | 10.33–11.04 | 7.61 | 6.52 | 6.21 | **0.00** |  |  |  |
| **6** | *A. loloensis* | 10.33–10.81 | 6.56 | 5.39 | 3.99 | 6.79 | **0.00** |  |  |
| **7** | *A. minutus* | 8.70–10.04 | 6.52–6.66 | 5.77–6.59 | 5.30–6.37 | 6.82–7.36 | 5.61–5.84 | **0.00** |  |
| **8** | *A. lifanensis* | 18.40–19.14 | 17.25 | 17.37 | 18.69 | 18.89 | 19.36 | 17.63–17.94 | **0.00** |

**Table 4. Uncorrected pairwise genetic distance (%) between members of the genus *Amolops* estimated from cyt*b* sequences**

|  | **Species** | 1 | 2 | 3 | 4 | 5 | 6 | 7 | 8 | 9 | 10 | 11 |
| --- | --- | --- | --- | --- | --- | --- | --- | --- | --- | --- | --- | --- |
| 1 | *Amolops cuongi* sp. nov. | **0–1.89** |  |  |  |  |  |  |  |  |  |  |
| 2 | *A. granulosus* | 8.02–10.01 | **0.00–0.35** |  |  |  |  |  |  |  |  |  |
| 3 | *A. mantzorum* ssp. | 9.91–11.53 | 7.30–7.44 | **0.00–0.23** |  |  |  |  |  |  |  |  |
| 4 | *A. mantzorum* | 8.46–10.03 | 6.72–7.05 | 2.82–3.32 | **0.00–0.38** |  |  |  |  |  |  |  |
| 5 | *A. jinjangensis* | 8.46–10.72 | 5.54–5.67 | 6.69–6.95 | 6.89–7.00 | **0.00** |  |  |  |  |  |  |
| 6 | *A.* cf. *mantzorum* | 8.46–10.29 | 7.31–7.82 | 3.31–3.46 | 3.06–3.61 | 7.35–7.57 | **0.00–0.25** |  |  |  |  |  |
| 7 | *A. minutus* | 8.28–10.46 | 7.08–7.22 | 7.35–7.61 | 7.87–8.39 | 5.31 | 7.49–7.70 | **0.00** |  |  |  |  |
| 8 | *A. loloensis* | 7.80–9.61 | 6.15–6.67 | 7.58–7.97 | 7.26–7.45 | 5.04–5.21 | 7.49–8.00 | 6.06–6.30 | **0.00–0.50** |  |  |  |
| 9 | *A. tuberodepressus* | 10.40–10.92 | 6.97–7.11 | 6.84–6.98 | 7.11–7.27 | 5.07 | 6.85–7.14 | 5.60 | 6.16–6.42 | **0.00** |  |  |
| 10 | *A. mantzorum xinduqiao* | 9.41–11.11 | 7.18–7.31 | 2.70–2.94 | 2.46–2.67 | 6.31 | 3.67–3.85 | 6.97 | 6.02–6.30 | 6.70 | **0.00** |  |
| 11 | *A. ailao* | 9.17–10.43 | 8.10–8.24 | 7.07 | 6.81–7.55 | 6.29 | 7.47–7.84 | 5.42 | 6.86–7.20 | 6.70 | 6.69 | **0.00** |
