# Supplementary material for: ﻿Amolops cuongi (Amphibia, Anura, Ranidae), a new species from the Hoang Lien Range, Vietnam
Source: Zookeys. 2025 Oct 22;1256:235–57. doi: 10.3897/zookeys.1256.158846 (PMC12572962; doi:10.3897/zookeys.1256.158846)
Supplement: Supplementary material 2 — Supplementary figures [file zookeys-1256-235_article-158846__-s002.doc]

***Amolops cuongi* (Amphibia: Anura: Ranidae), a new species from the Hoang Lien Range, Vietnam**

**Supplementary figures 1, 2**


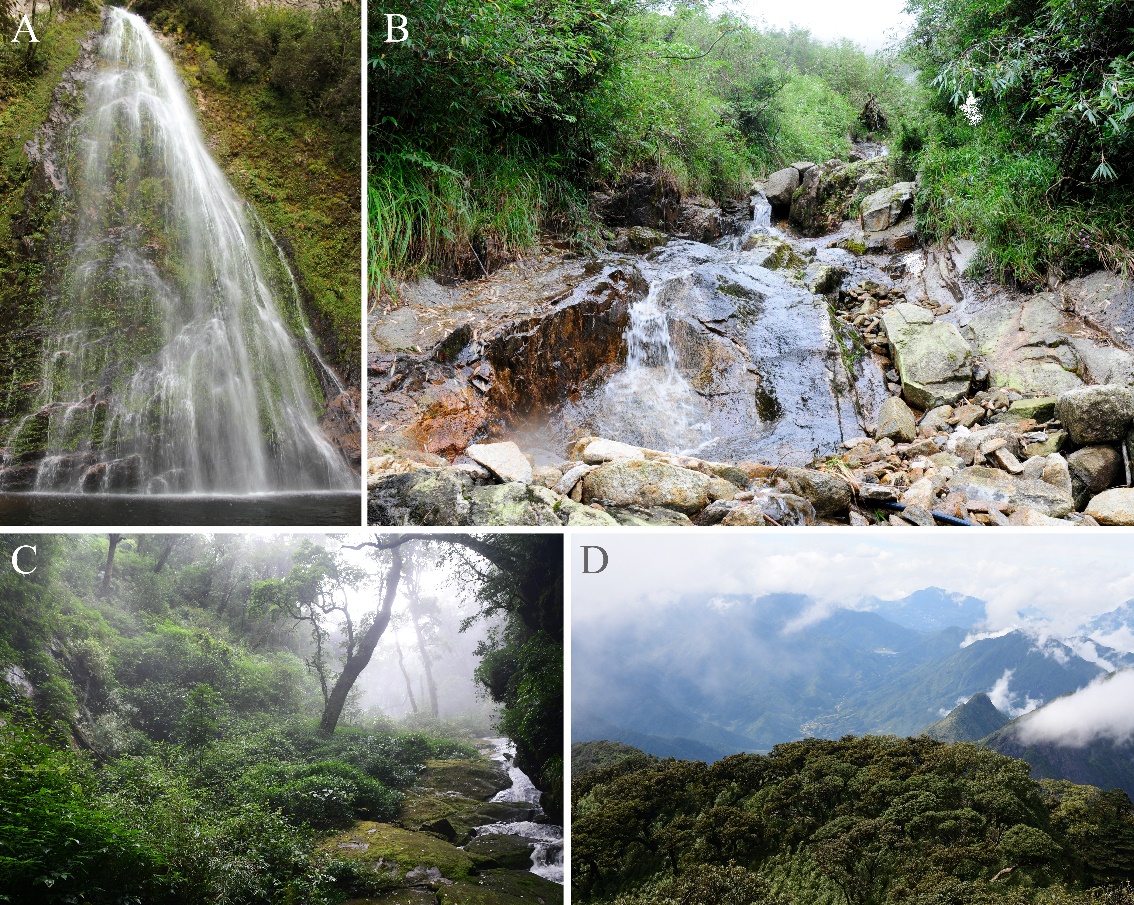


**Figure 1.** Habitat of *Amolops cuongi* sp. nov. in the Hoang Lien Range. (**A**) paratype collection site on Mount Fansipan, Tam Duong District, Lai Chau Province at 1928 m; (**B)** Collection site on Mount Fansipan, Lao Cai Province at 2650 m, (**C)** Collection site on Mount Nam Kang Ho Tao, Lao Cai Province at 2330 m (**D)** Macrohabitat, Mount Nam Kang Ho Tao, Lao Cai Province.


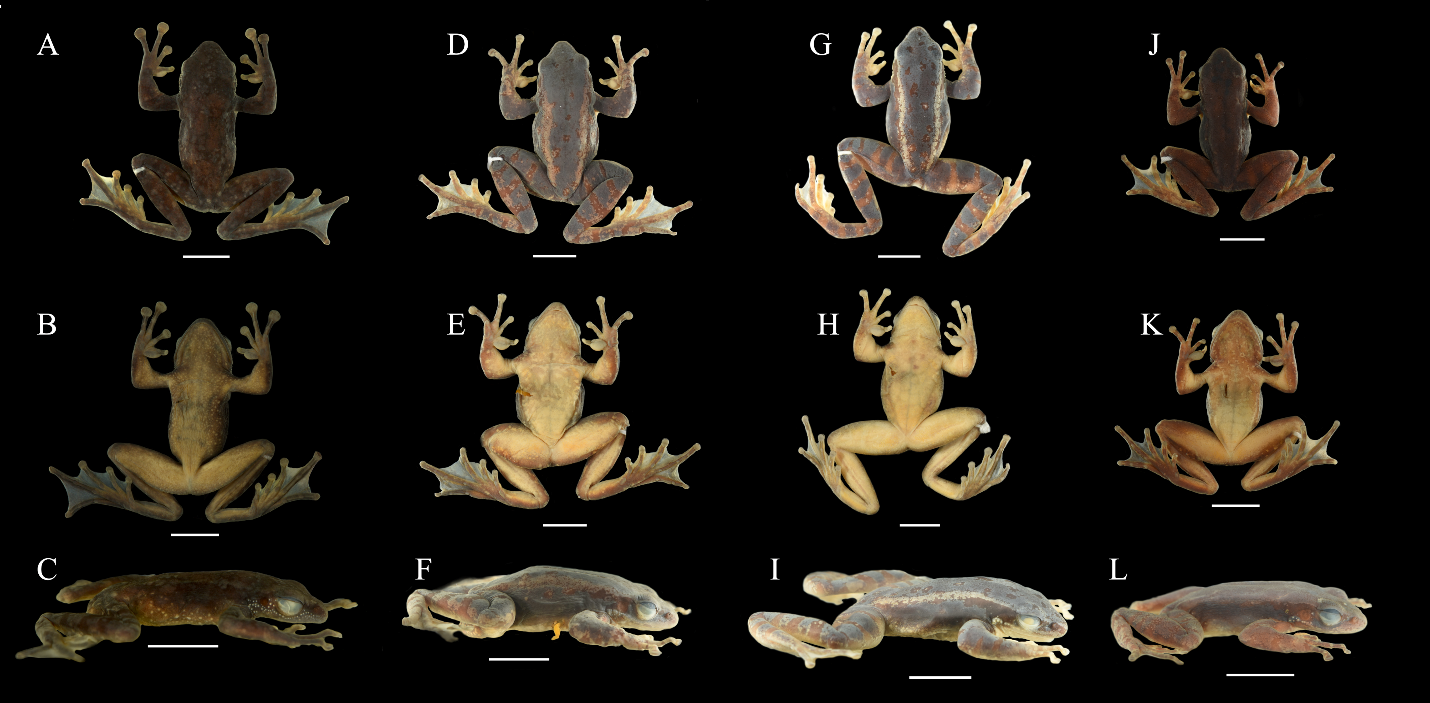


**Figure 2.** (**A-C**) *Amolops decorus* sp. nov. adult male paratype ILS H.3665 dorsal, ventral and lateral views in preservative. *Amolops minutus* dorsal, ventral and lateral views in preservative (**D-F**) adult male ILS H.3678; (**G-I**) adult male ILS H.3681; (**J-L**) adult male ILS H.3679. Scale bars 10 mm.
